# Modelling factors associated with therapeutic inertia in hypertensive patients: Analysis using repeated data from a hospital registry in West Africa

**DOI:** 10.1097/MD.0000000000031147

**Published:** 2022-12-09

**Authors:** Mahamadou Barro, Aristide Relwendé Yaméogo, Robert Darlin Mba, Rémi Kaboré, Germain Mandi, Désiré Lucien Dahourou, Patrice Zabsonré, Nicolas Méda, Juste Goungounga

**Affiliations:** a Institut de Recherche en Sciences de la Santé, Département de Biomédical/Santé Publique, Ouagadougou, Burkina Faso; b IDEES Le Havre, UMR CNRS 6266, Université du Havre, Normandie Université, Le Havre, France; c Service de cardiologie, Centre Hospitalier Universitaire Yalgado Ouédraogo, Ouagadougou, Burkina Faso; d UFR-SDS, Département de la Santé Publique; Université Joseph KI-ZERBO, Ouagadougou, Burkina Faso; e Aix Marseille Univ, INSERM, IRD, SESSTIM Sciences Économiques & Sociales de la Santé & Traitement de l’Information Médicale, Marseille, France; f, Bordeaux School of public health/Université de Bordeaux, Bordeaux, France; g Digestive Cancer Registry of Burgundy, Dijon University Hospital, Dijon, France; Unit 1231 Epidemiology and Clinical Research in Digestive Cancers, National Institute of Health and Medical Research, University of Burgundy-Franche Comte, Dijon, France; h Univ Rennes, EHESP, CNRS, Inserm, Arènes-UMR 6051, RSMS-U 1309, F-35000, Rennes, France; i Écoles Des Hautes Études en Santé Publique, Département METIS, 15 Avenue du Professeur Léon Bernard, CS 74312, 35043, Rennes Cedex, France.

**Keywords:** blood pressure variability, determinants, heterogeneity, hypertension, therapeutic inertia

## Abstract

The proportion of poorly controlled hypertensives still remains high in the general African population. This is largely due to therapeutic inertia (TI), defined as the failure to intensify or modify treatment in a patient with poorly controlled blood pressure (BP). The objective of this study was to identify the determinants of TI. We conducted a retrospective cohort study from March 2012 to February 2014 of hypertensive patients followed during 4 medical visits. The TI score was the number of visits with TI divided by the number of visits where a therapeutic change was indicated. A random-effects logistic model was used to identify the determinants of TI. A total of 200 subjects were included, with a mean age of 57.98 years and 67% men. The TI score was measured at 85.57% (confidence interval [CI] 95% = [82.41–88.92]). Measured individual heterogeneity was significantly significant (0.78). Three factors were associated with treatment inertia, namely the number of antihypertensive drugs (odd ratios [OR] = 1.27; CI = [1.02–1.58]), the time between consultations (OR = 0.94; CI = [0.91–0.97]), and treatment noncompliance (OR = 15.18; CI = [3.13–73.70]). The random-effects model performed better in predicting high-risk patients with TI than the classical logistic model (*P* value < .001). Our study showed a high TI score in patients followed in cardiology in Burkina Faso. Reduction of the TI score through targeted interventions is necessary to better control hypertension in our cohort patients.

## 1. Introduction

High blood pressure is responsible for significant cardiovascular morbidity and mortality in many countries. Nearly 600 million people, or 20% of the adult population, are affected worldwide. According to the World Health Organization, it is responsible for 9.4 million deaths per year worldwide due to its complications, 75% of which occur in developing countries.^[1]^ And if strategies to control this disease are not put in place, the number of hypertensive patients in the world will reach 1.56 billion in 2025, 2 thirds of which will be in developing countries.^[2]^

Sub-Saharan Africa has high prevalence of hypertension among adults aged 18 years and older, ranging from 16% to 40%.^[3]^ This prevalence of hypertension varies from country to country and by area of residence.^[4]^ A prevalence of 16.9% of the adult population aged 15 years and older has been reported in Ethiopia^[5]^ compared with 36.7% in Ghana.^[6]^ In urban areas, the prevalence was 23% in Benin^[7]^ compared to 54.6% in Ghana.^[8]^ In rural areas, the prevalence was 14.6% in Uganda^[9]^ and 44.5% in Nigeria.^[10]^

In Burkina Faso, the 2013 STEPS survey showed that among the population aged 25 to 64 years, the overall prevalence of hypertension was 17.6%. It was 24.8% in urban areas and 14.8% in rural areas.^[11,12]^ In the studies of Soubeiga et al and Yameogo et al prevalences of 18% and 29.6% were found respectively.

Thus, all the recommendations insist on the importance of managing hypertension and in particular on good control of blood pressure (BP). However, achieving BP control is not at all easy in practice. It remains insufficiently controlled according to most of the data in the literature, especially in the African population.^[13,14]^ The combination of multiple factors could explain these results, among which, the therapeutic inertia (TI) of practitioners has been identified as an important factor that has not improved in recent years. By definition, TI in the management of hypertension is the absence of intensification or modification of antihypertensive treatment in a patient with nonstandard BP figures.^[15–17]^ Unfortunately, in our setting, little is known about the factors related to TI. Yet, if the inertia rate were to be reduced from 85% to 70%, BP control could increase from 46% to about 65% in 1 year.^[15]^ The BP figure as well as the TI value varies at each visit. However, failure to take into account the variability of these data may lead to erroneous results, for example, an erroneous estimation of the factors associated with this pathology. This can impact medical decision making.

In this work, our objective is to determine the factors associated with TI in hypertensive patients received in outpatient cardiology consultation at the Yalgado Ouédraogo University Hospital (CHUYO) between March 2012 and February 2014.

## 2. Materials and methods

### 2.1. Type of study and study population

We conducted a retrospective cohort study between March 2012 and February 2014 with patients followed up in outpatient cardiology for hypertension who had at least 4 cardiology visits spaced at least 1 month apart. Included in the study were all patients seen in outpatient clinics for diagnosis of hypertension who were at least 15 years old. Patients who refused to give consent were not included in the study.

### 2.2. Sampling

Sampling was exhaustive for all patients who were eligible during the study period and who gave their consent. A data collection form was filled out by us using information from the patients’ health record and consultation registers. We used a methodological approach that took into account the repeated nature of the data. Indeed, longitudinal data allow us to study the evolution of a biological or clinical response for different individuals over time and the influence of the characteristics of the subjects on this evolution. When analyzing longitudinal data, the correlation and heterogeneity between measurements of a characteristic from the same subject must be considered.

### 2.3. Statistical analysis

The data processing and statistical analysis were done using R software in its version 3.5.3. After cleaning and formatting the data, we performed a descriptive analysis of the data. We calculated the frequency of TI, TI scores and justification of TI. We have removed the subjects with missing data.

We then individually tested the association between each of our clinical and sociodemographic characteristic variables with TI using the random effects mixed logistic model, due to the repeated structure of the data. Variables in which 1 modality was statistically associated at the 30% threshold (*P* value = .3) with TI were retained as potential candidates’ covariates for the multivariate analysis.

Finally, a final model was fitted with the variables retained in the bivariate analysis. This was a random-effects mixed logistic model by considering individual variability in BP during visits. The Wald test was used to measure the significance of the coefficients and were considered significant if the *P* value was <.05. We also measured the correlation between the explanatory variables. These results were compared to those of a fixed-effects logistic model to measure the impact of considering the individual heterogeneity of the different repeated measures. We used the Akaike Information Criterion to measure the quality of our mixed model compared to the fixed effects logistic model.^[18]^ We then calculated and compared the area under the receiver operator characteristic (ROC) curve (AUC) of these 2 models using the DeLong’s test, in order to evaluate their predictive performance in terms of prediction of the occurrence of TI for an individual, as a clinical decision-making tool. We also used the accuracy of these 2 models, that is, the fraction of individuals correctly classified with a high risk of TI, as well as their sensitivity and specificity values to assess their ability to discriminate between subjects at high risk of TI. ROC curves and AUC were implemented using the R package pROC.^[19]^

### 2.4. Ethical review

The director of the teaching university hospital CHUYO were fully informed of the objectives and progress of the study. Oral consent was sought and obtained from patients who still had visits. The contact of the investigators and the ethics committee were communicated to them. The confidentiality of each participant was strictly respected. To this end, an anonymity number was assigned to each participant. At the regulatory level, the authorization of the medical commission of the teaching university hospital CHUYO was requested and obtained before the start of the data collection.

## 3. Results

### 3.1. Sociodemographic and clinical characteristics

A total of 200 patients were included in our study. We had a total of 2 patients who declined to participate in the study. Approximately 67% of the patients were women. The mean age was 57.98 years with extremes of 23 and 99 years. Two thirds of the patients lived in Ouagadougou (67.65%). The majority of patients (63%) were married and illiterate people represented 47% of patients (Table 1). Regarding clinical characteristics, 18% of patients were diabetic and 24% were obese. Stroke had affected 17% of our subjects and congestive heart failure was present in 8%. The mean number of molecules in our study was 2.2; 95% confidence interval (CI) = (2.07–2.32) with extremes of 1 and 5 molecules. The mean duration of hypertension in our study was 8.53 years; 95% CI = (7.70–9.37) with extremes of 1 and 34 years (Table 2).

**Table 1:** Distribution of hypertensive cardiology outpatients at CHUYO between March 2012 and February 2014 by sociodemographic characteristics.

Features	Numbers (N = 200)	Percentage (%)
**Gender**		
Male	134	67
Female	66	33
**Age (yrs**)		
<25	2	1
[25; 35]	4	2
[35; 45]	24	12
[45; 55]	47	23.5
[55; 65]	60	30
≥65	63	31.5
**Average (standard deviation**)	57.98 yrs (12.96)	
**Place of residence**		
Ouaga	153	67.65
Outside Ouaga	47	23.5
**Marital status**		
Married	126	63
Widower	58	29
Concubinage	11	5.5
Single	5	2.5
**Level of education**		
Illiterate	94	47
Primary	31	15.5
Secondary	71	35.5
Superior	4	2
**Profession**		
Employee	74	37
Cultivator	16	8
housewife	84	42
Informal	26	13

CHUYO = Yalgado Ouedraogo teaching Hospital.

**Table 2:** Distribution according to clinical characteristics of hypertensive outpatients in cardiology at CHUYO between March 2012 and February 2014.

Features	Numbers N = 200	Percentage (%)
**Risk factors for cardiovascular**		
Diabetes	36	18
Alcohol	2	1
Tobacco	16	8
Sedentary lifestyle	14	7
Obesity	48	24
Dyslipidemia	25	12.5
**Compliance with treatment**	6	3
**Complications of hypertension**		
Metabolic syndrome	8	4
Global heart failure	16	8
Myocardial infarction	6	3
Stroke	34	17
Renal failure	8	4
**Number of antihypertensive drugs**		
Monotherapy	49	24.5
Dual therapy	80	40
Tritherapy	55	27.5
Quadritherapy and more	16	8
**Evolution of hypertension**		
[0-5 yrs]	47	23.5
[5-10 yrs]	82	41
[10-15 yrs]	39	19.5
[15-20 yrs]	21	10.5
More than 20 yrs	11	5.5

CHUYO = Yalgado Ouedraogo teaching Hospital.

### 3.2. Frequency and scores of TI

The mean TI score or TI score of all consultations was 85.9% CI = (82.41; 88.92) with variations from 80.56% to 90.47%. The mean justification score or J score for all consultations was 86% CI = (82.83; 89.74) with variations from 91.67% to 79.31% (Table 3).

**Table 3:** Measures of therapeutic inertia scores in the cardiology outpatient hypertensive population at CHUYO between March 2012 and February 2014.

Consultations	TI score (%)	CI (95%)	J score (%)	CI (95%)
**Global**	**85.90**	**[82.41-88.92]**	**86.57**	**[82.83-89.74]**
Consultation 1	88.52	[81.49-93.58]	91.67	[84.76-96.11]
Consultation 2	83.74	[76.01-89.77]	86.41	[78.24-92.36]
Consultation 3	90.43	[83.52-95.12]	87.5	[79.57-93.17]
Consultation 4	80.56	[71.82-87.54]	79.31	[69.28-87.25]

CHUYO = Yalgado Ouedraogo teaching Hospital, CI = confidence interval, TI = therapeutic inertia.

### 3.3. Factors associated with TI

Two determinants were associated with an increase in TI, namely the number of antihypertensive drugs odd ratios (OR) = 1.27 ({IC95% [1.02–1.58]}), and nonadherence to treatment (OR = 15.18 {IC95% [3.13–73.70]}). Time between visits was the only determinant associated with decreased treatment inertia, OR = 0.94 {IC95% [0.91–0.97]} (Table 4). The measured correlation between the independent variables in the multivariate regression was low. All correlation coefficients were <30% (Table 5).

**Table 4:** Factors associated with therapeutic inertia after mixed random-effects logistic regression and a simple logistic regression.

		Mixed		Simple	
Type of effect	Variables (Reference)	OR (CI)	*P* value	OR (CI)	*P* value
Fixed	Marital status (Married)	1		1	
	Concubinage	0.62 [0.26-1.48]	.27	0.66 [0.34-1.27]	.22
Fixed	Single	0.47 [0.14-1.65]	.24	0.50 [0.19-1.31]	.16
	Widower	0.76 [0.49-1.16]	.20	0.79 [81.49-93.58]	.15
Fixed	Tobacco (No)	1.72 [0.84-3.46]	.13	1.62 [0.57-1.09]	.09
	Obesity (No)	0.69 [0.44-1.08]	.10	0.72 [0.93-2.77]	.06
Fixed	Heart failure (No)	1.52 [0.74-3.16]	.25	1.43 [0.51-1.02]	.20
	Duration of hypertension	0.98 [0.94-1.01]	.16	0.98 [0.83-2.51]	.11
Fixed	Number of anti-hypertensive drugs	1.27 [1.02-1.58]	**.02**	1.23 [1.05-1.46]	**.01**
	Time between consultations	0.94 [0.91-0.97]	**.001**	0.94 [0.92-0.97]	**.001**
Fixed	Compliance with treatment (Yes)	15.18 [3.13-73.70]	**.001**	11.13 [2.53-49.40]	**.002**
**Random**	**Consultation***(individual variability*)	*0.78 (0.63)			
	**AIC**	**1058.9**		**1072.2**	

AIC = Akaike information criterion, CI = confidence interval, OR = odd ratios.

*P* value in bold: variables associated with IT; * variance (standard error)

**Table 5:** The correlation measured in the multivariate analysis for the fixed-effects variables.

Variables	Concubinage	Single	Widower	Tobacco	Obesity	HF	Hypertension	Anti-hypertensive drugs	Medical appointment
Single	0.06								
Widower	0.13	0.10							
Tobacco	-0.08	-0.09	0.09						
Obesity	-0.03	-0.009	0.02	0.03					
Heart failure	0.03	-0.19	-0.124	0.009	0.04				
Duration of hypertension	0.06	0.13	-0.16	-0.05	-0.11	0.05			
Number of anti-hypertensives	0.09	-0.08	0.03	0.10	-0.11	-0.10	-0.15		
Time between consultations	0.04	0.10	-0.01	-0.01	-0.02	-0.02	0.002	-0.05	
Compliance with treatment	-0.07	0.002	-0.04	0.02	-0.003	0.01	0.02	0.01	-0.063

HF = heart failure.

### 3.4. Prediction of the occurrence of TI

**Figure 1.**
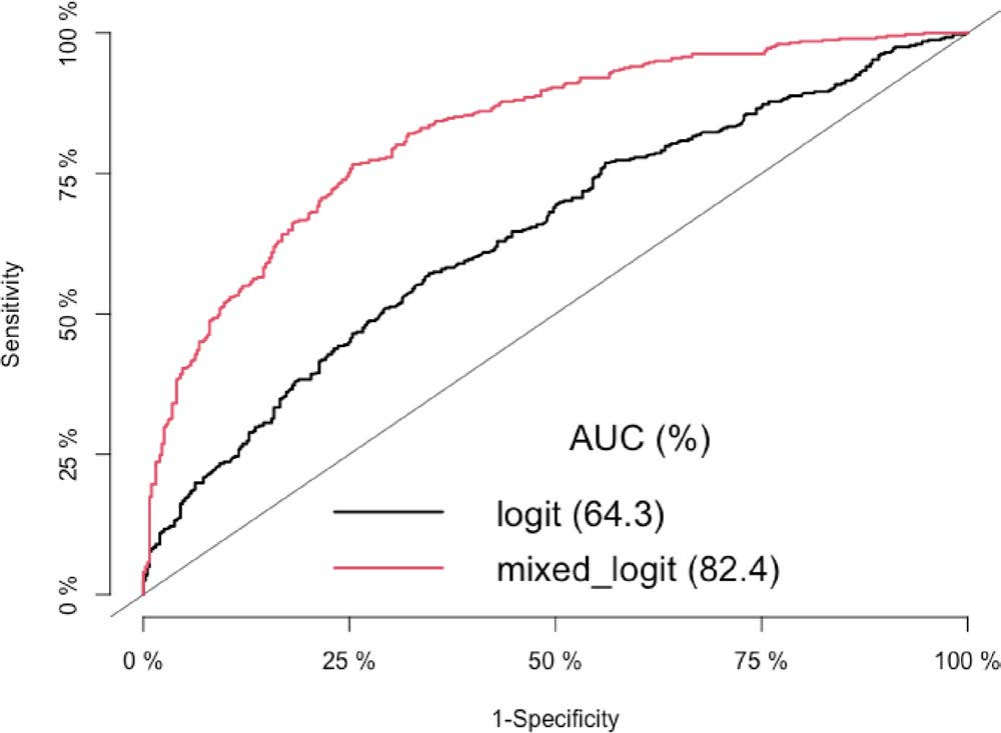
Logit: the classical (black line) logistic model using the area under the curve (AUC). Mixed logit: the random-effects logistic model (red line) using the AUC.

ROC curve for predicting of the occurrence of TI with the classical and the random-effects logistic regression model are shown in Figure 1. In terms of the AUC values the random-effects logistic model performed significantly better than classical logistic model (AUC and their 95% CI: 82.4 [79.56; 85.23] vs 64.3 [60.51; 68.10]; *P* value < 2.2 e-16). The optimal cut points to predict TI were 0.49 versus 0.51 with accuracy of 0.75 (sensitivity = 76.62%; specificity = 74.62%) versus 0.61 (sensitivity = 57.21%; specificity = 65.33%) for the random-effects logistic and the classical logistic model respectively.

## 4. Discussion

The objective of this study was to estimate the scores and identify the determinants of TI in patients followed up in outpatient cardiology for hypertension who had at least 4 cardiology consultations spaced at least 1 month apart in Burkina. The limit of our study is the lack of collection of cardiovascular events occurring during follow-up.

In our study, the TI score in hypertensive patients was 85.9%. This score could be explained in our context by late initiation of treatment. In many diseases, the earlier the treatment is initiated, the higher the success of the treatment.^[19]^ This high score confirms the high frequency mentioned above in our study and implies at the same time a probable poor management of hypertension in our context. Therefore, if there is a finding of TI, we can expect a higher risk of complications related to the lack of control of their hypertension. In addition to the consequences for the patient, TI can have major consequences for society, since hypertension concerns the dynamic fringe of the population and the patients are in most cases heads of families.^[19]^ This result seems to be very close to those of the literature,^[15,20]^ respectively 86.9%, 84%, and 78%. We used the same definition of TI as these studies. However, our score was higher than those of Guthrie et al (41%)^[21]^ and Pretorean et al (8.3%).^[22]^ These discrepancies may be explained by the use of different definitions of the concept of TI. Our definition does not take into account the “adapted rationale” explaining the absence of therapeutic modification and BP thresholds adapted to comorbidities; this could explain a higher level of detection of TIs in our study. It should be emphasized that the Pretorean et al study took place in an expert center for hypertension and vascular disease that is more sensitive to TI than elsewhere.

The literature^[23,24]^ showed much lower TI justification scores than our results (86.57%) of 37.7% and 59.4%, respectively. This could be explained not only by the type of study but also by the study population. Indeed, the study by Bencherif et al was an observational study including diabetic and/or hypertensive patients. In addition, we did not have the same definition of TI in our different studies. In their study, TI occurred when a BP value was higher than 130/80 mm Hg in cases of diabetes or chronic renal failure, and 140/90 mm Hg in other cases. The Crowley et al study was an intervention study of 593 veterans. The high TI score found in our study could be explained by the fact that some practitioners would give inappropriate justifications for not changing the treatment of patients. Indeed, this score is used to evaluate, after analysis, patient-centered practice. Any medical practice must be adapted to the patient’s history and environment. The physician thus justifies not modifying the treatment when, if the recommendations were applied “rigorously,” modification would be indicated. This exacerbates the effects of the TI by increasing the number of uncontrolled and non-compliant patients.

Among the determinants investigated in our study, 3 are associated with an increase or decrease in TI, namely the number of antihypertensive drugs, the time between consultations, and noncompliance with treatment.

There was more TI in patients on multiple therapy. Indeed, the increase of 1 molecule in the patient’s treatment increased the risk of TI by 27% (*P* = .002). According to a review of the literature,^[25]^ 25% of the authors found a significant decrease in TI with the number of antihypertensive drugs and also 25% found an increase, while half found no association between the 2. The studies by Crowley et al (OR for each additional treatment: 1.24; *P* < .05) and Turner et al with an increasing OR from 1.16 (1 anti-hypertensive drug treatment) to 2.38 (≥4 1 anti-hypertensive drug treatments) with *P* < .001 have results close to ours. However, our results diverge with those of Okonofua et al (OR for each additional treatment: 0.52; *P* < .0001) and Kerr et al (Association not significant; *P* > .05). This discrepancy could be explained by the fact that the Kerr et al study was prospective with a mixed population of diabetic patients and treatment progress was assessed for 1 visit per patient. The study by Okonofua et al was retrospective with a longer follow-up and a larger sample size than in our study. The identification of this association should challenge the practitioner to consider factors such as patient preferences and values, medication burden, and other medical or personal circumstances. It will help to reduce TI and its consequences.

Regarding the time between consultations, there was less TI in patients with long durations between consultations. Indeed, increasing the time between consultations by 1 month decreased the risk of inertia by 6% (OR = 0.94 *P* < .001). Our results differed from those of the literature such as van Bruggen et al,^[26]^ Hicks et al,^[27]^ Redon et al,^[20]^ and Turner et al.^[28]^ However, in all these studies, the frequency concerned the number of consultations. The only study in which this notion referred to the time elapsed between 2 consultations, as in our study, was that of Andrade et al.^[29]^ In his study, there was no difference in TI according to the time elapsed since the previous consultation. This discrepancy with our results could be attributed to the design of the different studies. The study by Andrade et al included subjects aged between 45 and 84 years, whereas the risk of TI increases with age. The evidence of this association supports the interest of a structured and regular continuous medical checkup. It should encourage health care decision-makers to take normative measures for visits and to define with practitioners an adapted and personalized care pathway for patients. If the patient is stable and BP is controlled, then appointments should be spaced at a maximum of 2 contacts per year so that the patient does not experience his or her disease as a burden. Otherwise, appointments should be brought closer together to improve patient compliance with treatment and to provide therapeutic education.

There was more TI in patients who did not adhere to their treatments. Indeed, a patient who did not adhere to his or her treatment was 15 times more likely to have TI than a patient who normally adhered to his or her treatment (*P* < .001). Our results diverge from those of the literature. Only the Rose et al study^[30]^ found an association between TI and treatment non-adherence. The other studies did not find an association, that is, 83% of the literature according to Bien et al.^[25]^ Indeed, studies by Bolen et al,^[31]^ Manze et al^[32]^ and Heisler et al^[33]^ are some of them. These discrepancies could be explained by several elements. First, this adhesion has been defined in different ways depending on the studies. Heisler et al defined it as the proportion of days when the patient should have taken the medication but had no medication available (called the “refill gap”). Bolen et al used an algorithm developed by Steiner et al that integrated the number of available medications and the number of days to treat. In the study by Manze et al it was an electronic monitoring device medication events monitoring system. According to a qualitative study,^[34]^ in 64% of the cases, treatment options were limited due to problems with the cost of treatment relative to patient income and adverse events. The evidence of this association should challenge health care decision-makers to take measures to make treatments geographically and financially available to patients. This is to reduce TI, which has disastrous consequences on patients and societies in terms of health and socio-economics.

In our study we could not establish associations between TI and variables such as gender, age, income, smoking, dyslipidemia and diabetes. In the same perspective, according to a literature review study^[25]^ respectively 61%, 64%, 100%, 100%, 40%, and 46% of the literature before 2016 had found no association between TI and the respective variables of gender, age, income, and smoking. Depression, dyslipidemia, and diabetes were variables that 67%, 60%, and 54% of the literature found to be associated with treatment inertia, respectively.^[25]^ Study design, study populations, definitions of TI, and measurement of TI over a period or by visit were the main reasons for differences in the results of these studies (decreased, increased, or no association).

To identify the determinants of physician decision making in terms of TI during follow-up, we used a random-effects mixed logistic regression. This method takes into account the correlation and heterogeneity between repeated measurements of the same characteristic (e.g., BP) observed in an individual. The interest of this data type-specific modeling shows why the OR are then underestimated using the classical logistic regression method, which does not take into account the inter- and intra-individual variability, compared with the logistic mixed model. Moreover, the interest of the mixed logistic regression rather than the simple logistic regression can be clarified by using statistical criteria such as the Akaike information criterion or a likelihood ratio test. However, for predicting the high risk of TI, ROC curves allow to evaluate the more appropriate approach for decision making.

## 5. Conclusion

The results of our study provide important information about factors that contribute to TI in clinical management of hypertension patients in Burkina Faso. Efforts should be pursued to disseminate the concept of TI and decrease its intensity. However, given the complexity of managing the hypertensive patient, eliminating treatment inertia may not guarantee better management, but the development of decision support systems will improve compliance with hypertension management recommendations and reduce the risk of TI among health workers. When considering intensifying antihypertensive therapy, practitioners should consider factors such as patient preferences and values, drug burden, and other medical or personal circumstances of the patient. Further work, including qualitative studies involving practitioners and patients, will be needed to further clarify the factors associated with TI, including situations of justified TI that correspond to an appropriate medical decision for the patient. Such an innovative approach combining machine learning and clinical diagnostic procedures will make any prediction tool for high-risk situations of TI useful and efficient.

## Acknowledgments

The authors thank the Reviewers and the Editor for the revision of the submitted version of this manuscript.

## Author contributions

**Conceptualization:** Mahamadou Barro, Juste Goungounga, Aristide Relwendé Yameogo, Germain Mandi, Patrice Zabsonre, Nicolas Meda.

**Data curation:** Mahamadou Barro, Juste Goungounga, Aristide Relwendé Yameogo, Germain Mandi.

**Formal analysis:** Mahamadou Barro, Juste Goungounga, Aristide Relwendé Yameogo, Robert Darlin, Remi Kabore, Désiré Lucien Dahourou.

**Investigation:** Mahamadou Barro, Juste Goungounga, Aristide Relwendé Yameogo.

**Methodology:** Mahamadou Barro, Juste Goungounga, Aristide Relwendé Yameogo, Remi Kabore, Désiré Lucien Dahourou, Nicolas Meda.

**Project administration:** Mahamadou Barro, Aristide Relwendé Yameogo, Germain Mandi, Patrice Zabsonre, Nicolas Meda.

**Supervision:** Mahamadou Barro, Juste Goungounga, Aristide Relwendé Yameogo.

**Validation:** Juste Goungounga, Aristide Relwendé Yameogo.

**Visualization:** Juste Goungounga.

**Writing – original draft:** Mahamadou Barro, Juste Goungounga, Aristide Relwendé Yameogo, Robert Darlin, Remi Kabore, Désiré Lucien Dahourou.

**Writing – review & editing:** Mahamadou Barro, Juste Goungounga, Aristide Relwendé Yameogo.

## References

[1] World Health Organization. A global brief on hypertension: silent killer, global public health crisis: World Health Day 2013. Geneva, Switzerland: World Health Organization. 2013.

[2] Kearney PM Whelton M Reynolds K. Global burden of hypertension: analysis of worldwide data. Lancet Lond Engl. 2005;365:217–23. 10.1016/S0140-6736(05)17741-1 15652604

[3] Houehanou C Amidou S Preux P-M. Hypertension in sub-Saharan Africa. JMV-J Médecine Vasc. 2018;43:87.

[4] Hendriks ME Wit FWNM Roos MTL. Hypertension in Sub-Saharan Africa: cross-sectional surveys in four rural and urban communities. Atashili J, éditeur. PLoS One. 2012;7:e32638. 22427857 10.1371/journal.pone.0032638 PMC3299675

[5] Bonsa F Gudina E Hajito K. Prevalence of hypertension and associated factors in Bedele Town, Southwest Ethiopia. Ethiop J Health Sci. 2014;24:21. 24591795 10.4314/ejhs.v24i1.3 PMC3929924

[6] Ghana Health Service. uSurvey of chronic noncommunicable diseases and their risk factors. 2006. Google Scholar [Internet]. 2006. Available at: https://journals.plos.org/plosone/article?id=10.1371/journal.pone.0020316. [Access date 20 September, 2019].

[7] Sodjinou R Agueh V Fayomi B. Obesity and cardio-metabolic risk factors in urban adults of Benin: relationship with socio-economic status, urbanisation, and lifestyle patterns. BMC Public Health. 2008;8:84. 18318907 10.1186/1471-2458-8-84 PMC2315643

[8] Addo J Agyemang C Smeeth L. A review of population-based studies on hypertension in Ghana. Ghana Med J. 2012;46(2 Suppl):4–11. 23661811 PMC3645150

[9] Kotwani P Kwarisiima D Clark TD. Epidemiology and awareness of hypertension in a rural Ugandan community: a cross-sectional study. BMC Public Health. 2013;13:1151. 24321133 10.1186/1471-2458-13-1151 PMC3890617

[10] Ahaneku G Osuji C Anisiuba B. Evaluation of blood pressure and indices of obesity in a typical rural community in eastern Nigeria. Ann Afr Med. 2011;10:120. 21691018 10.4103/1596-3519.82076

[11] Ministry of Health, Burkina Faso. Report of the national survey on the prevalence of major common risk factors for non-communicable diseases in Burkina Faso, STEPS survey 2013. 2014. Available at: https://www.who.int/ncds/surveillance/steps/BurkinaFaso_2013_STEPS_Report.pdf

[12] Soubeiga JK Millogo T Bicaba BW. Prevalence and factors associated with hypertension in Burkina Faso: a countrywide cross-sectional study. BMC Public Health. 2017;17:64. 28077112 10.1186/s12889-016-3926-8 PMC5225558

[13] Chobanian AV Bakris GL Black HR. The Seventh Report of the Joint National Committee on Prevention, Detection, Evaluation, and Treatment of High Blood Pressure: the JNC 7 report. JAMA 1979. 2003;289:2560–72. 10.1001/jama.289.19.2560 12748199

[14] Blacher J Halimi J-M Hanon O. Management of arterial hypertension in adults: 2013 guidelines of the French society of arterial hypertension. Sang Thromb Vaiss. 2013;9-10:297–305.

[15] Okonofua EC Simpson KN Jesri A. Therapeutic inertia is an impediment to achieving the Healthy People 2010 blood pressure control goals. Hypertens Dallas Tex 1979. 2006;47:345–51. 10.1161/01.HYP.0000200702.76436.4b 16432045

[16] Lebeau JP Cadwallader JS Aubin-Auger I. The concept and definition of therapeutic inertia in hypertension in primary care: a qualitative systematic review. BMC Fam Pract. 2014;15:130. 24989986 10.1186/1471-2296-15-130 PMC4094689

[17] Phillips LS Twombly JG. It’s time to overcome clinical inertia. Ann Intern Med. 2008;148:783783. 10.7326/0003-4819-148-10-200805200-00011 PMC3685435 18490691

[18] Aggrey SE. Logistic nonlinear mixed effects model for estimating growth parameters. Poult Sci. 2009;88:276–80. 19151340 10.3382/ps.2008-00317

[19] Robin X Turck N Hainard A. pROC: an open-source package for R and S+ to analyze and compare ROC curves. BMC Bioinf. 2011;12:77. 10.1186/1471-2105-12-77 PMC3068975 21414208

[20] Redón J Coca A Lázaro P. Factors associated with therapeutic inertia in hypertension: validation of a predictive model. J Hypertens. 2010;28:1770–7. 20531224 10.1097/HJH.0b013e32833b4953

[21] Guthrie B Inkster M Fahey T. Tackling therapeutic inertia: role of treatment data in quality indicators. BMJ. 2007;335:542–4. 17855323 10.1136/bmj.39259.400069.AD PMC1976517

[22] Pretorean T Claisse G Delsart P. [A specific questionnaire to evaluate therapeutic inertia in hypertensive patients: a pilot study]. J Mal Vasc. 2014;39:4–13. 24119421 10.1016/j.jmv.2013.09.001

[23] Ford ES Ajani UA Croft JB. Explaining the decrease in U.S. deaths from coronary disease, 1980-2000. N Engl J Med. 2007;356:2388–98. 17554120 10.1056/NEJMsa053935

[24] Crowley MJ Smith VA Olsen MK. Treatment intensification in a hypertension telemanagement trial: clinical inertia or good clinical judgment? Hypertension. 2011;58:552–8. 21844490 10.1161/HYPERTENSIONAHA.111.174367

[25] Bien B. Determinants of therapeutic inertia in the ambulatory treatment of cardiovascular risk factors: a review of the literature [Practice Thesis]. [France]: Pierre and Marie Curie University (Paris). UFR de médecine Pierre et Marie Curie. 2015.

[26] Van Bruggen R Gorter K Stolk R. Clinical inertia in general practice: widespread and related to the outcome of diabetes care. Fam Pract. 2009;26:428–36. 19729401 10.1093/fampra/cmp053

[27] Hicks PC Westfall JM Van Vorst RF. Action or inaction? Decision making in patients with diabetes and elevated blood pressure in primary care. Diabetes Care. 2006;29:2580–5. 17130188 10.2337/dc06-1124

[28] Turner BJ Hollenbeak CS Weiner M. Effect of unrelated comorbid conditions on hypertension management. Ann Intern Med. 2008;148:578–86. 18413619 10.7326/0003-4819-148-8-200804150-00002

[29] Andrade SE Gurwitz JH Field TS. Hypertension management: the care gap between clinical guidelines and clinical practice. Am J Manag Care. 2004;10(7 Pt 2):481–6. 15298234

[30] Rose AJ Berlowitz DR Manze M. Intensifying therapy for hypertension despite suboptimal adherence. Hypertens Dallas Tex 1979. 2009;54:524–9. 10.1161/HYPERTENSIONAHA.109.133389 PMC2739677 19581506

[31] Bolen SD Samuels TA Yeh H-C. Failure to intensify antihypertensive treatment by primary care providers: a cohort study in adults with diabetes mellitus and hypertension. J Gen Intern Med. 2008;23:543–50. 18219539 10.1007/s11606-008-0507-2 PMC2324132

[32] Manze M Rose AJ Orner MB. Understanding racial disparities in treatment intensification for hypertension management. J Gen Intern Med. 2010;25:819–25. 20386998 10.1007/s11606-010-1342-9 PMC2896595

[33] Heisler M Hogan MM Hofer TP. When more is not better: treatment intensification among hypertensive patients with poor medication adherence. Circulation. 2008;117:2884–92. 18506011 10.1161/CIRCULATIONAHA.107.724104

[34] Cotton A Aspy CB Mold J. Clinical decision-making in blood pressure management of patients with diabetes mellitus: an oklahoma physicians resource/research network (OKPRN) study. J Am Board Fam Med. 2006;19:232–9. 16672676 10.3122/jabfm.19.3.232

